# Invasive Asian water moss (*Salvinia cucullata*) biochar modulates selected steviol glycoside biosynthesis-related gene expression and drought-associated physiological responses in Stevia

**DOI:** 10.3389/fpls.2026.1859834

**Published:** 2026-07-10

**Authors:** Piyachat Sunanta, Pasin Norkum ai, Patipon Teerakitchotikan, Tibet Tangpao, Nguyen Vu Linh, Luu Tang Phuc Khang, Giancarlo Colelli, Sarana Rose Sommano

**Affiliations:** 1 Multidisciplinary Research Institute, Chiang Mai University, Chiang Mai, Thailand; 2 Plant Bioactive Compound Laboratory, Faculty of Agriculture, Chiang Mai University, Chiang Mai, Thailand; 3 Center of Omics for High-Value Agriculture (CMUAgOmics), Faculty of Agriculture, Chiang Mai University, Chiang Mai, Thailand; 4 Department of Plant and Soil Sciences, Faculty of Agriculture, Chiang Mai University, Chiang Mai, Thailand; 5 Department of Animal and Aquatic Sciences, Faculty of Agriculture, Chiang Mai University, Chiang Mai, Thailand; 6 Dipartimento di Scienze Agrarie, degli Alimenti e dell’Ambiente, Università di Foggia, Foggia, Italy

**Keywords:** biosynthesis-related gene, medicinal plant, plant-based sweetener, qPCR, soil amendment

## Abstract

**Introduction:**

Drought stress can impair the growth and alter the metabolic profile of *Stevia rebaudiana*, thereby affecting the production of commercially important steviol glycosides. Biochar is increasingly used as a sustainable soil amendment to improve plant performance under abiotic stress. However, its effects on stevia physiology and steviol glycoside-related gene expression remain insufficiently understood. This study evaluated the effects of biochar derived from the invasive aquatic weed *Salvinia cucullata* on growth traits, antioxidant-related biochemical responses, steviol glycoside accumulation, and the expression of selected biosynthesis-related genes in stevia grown under different water-holding capacities (WHC).

**Methods:**

Stevia plants were grown under varying WHC levels ranging from 100% to 45%, with and without S. cucullata-derived biochar amendment. Plant growth traits, biomass-related parameters, antioxidant-related biochemical responses, steviol glycoside contents, and the expression of selected steviol glycoside biosynthesis-related genes were evaluated. Quantitative PCR was used to assess the transcript abundance of selected genes involved in steviol glycoside biosynthesis.

**Results:**

Biochar amendment was associated with increased transcript abundance of KAH and UGT76G1, together with a modest reduction in UGT74G1 expression. These transcriptional patterns were accompanied by decreased stevioside content and relatively stable rebaudioside A levels. Morphological responses were mainly reflected in reduced vertical growth and increased lateral shoot development under some water-limited conditions, whereas leaf area, total biomass, and root weight were not consistently improved. Biochar-containing treatments also affected antioxidant-related traits, including higher total flavonoid content, gallic acid concentration, and DPPH radical scavenging activity under specific WHC conditions, while quercetin tended to be higher in the control treatment.

**Conclusion:**

*S. cucullata*-derived biochar was associated with selective changes in growth architecture, antioxidant-related metabolism, and the expression of selected genes related to steviol glycoside biosynthesis. These findings suggest that biochar may influence stevia responses to water limitation through both physiological and metabolic pathways. Further studies integrating soil chemistry, stress signaling markers, and broader transcriptomic or metabolomic analyses are required to clarify the underlying soil-plant and molecular mechanisms.

## 1 Introduction

The increasing frequency and severity of drought events pose a growing threat to agricultural productivity, particularly for high-value specialty crops that are sensitive to water limitation. *Stevia rebaudiana*, a perennial herb in the Asteraceae family, is widely cultivated for steviol glycosides, a group of high-intensity, low-calorie sweeteners with considerable commercial value (Zhang and Zhang, 2025). However, stevia is relatively sensitive to water deficit, and drought stress reduces plant growth and alter the accumulation of its major bioactive compounds. Previous studies have shown that drought stress causes a range of physiological and biochemical changes in *S. rebaudiana*, including visible wilting, reduced biomass accumulation, impaired photosynthetic performance, and altered secondary metabolism. These responses are commonly associated with oxidative stress and the formation of reactive carbonyl species, including lipid-derived aldehydes, which may contribute to cellular damage, chlorophyll degradation, and stress-related senescence when detoxification systems are insufficient. Nurbekova et al. (2024), showed in *Arabidopsis thaliana* that aldehyde oxidase-related detoxification influenced the accumulation of reactive aldehydes under oxidative stress conditions. In stevia, drought stress has also been associated with altered expression of genes involved in steviol glycoside biosynthesis, including kaurenoic acid hydroxylase (KAH) and UDP-glycosyltransferase (UGT) genes. Transcriptional responses may contribute to changes in important steviol glycosides, including rebaudioside A and stevioside (Ghaderi et al., 2025; Schiatti-Sisó et al., 2023; Wang et al., 2023). Steviol glycoside biosynthesis originates from the plastidial methylerythritol 4-phosphate (MEP) pathway and proceeds through downstream diterpenoid and glycosylation reactions involving KAH and UGTs. These enzymes contribute to the final composition of steviol glycosides in stevia (Ghose et al., 2022; Kim et al., 2015).

Sustainable soil amendments have received increasing attention as practical approaches for improving plant performance under abiotic stress. Biochar is of particular interest because it can enhance soil water retention, nutrient availability, and other physicochemical properties that support plant growth under water-limited conditions (Haider et al., 2020; Li et al., 2021). At the same time, invasive aquatic macrophytes such as *Salvinia cucullata* (Asian water moss) represent abundant but underutilized biomass resources. Conversion of this invasive plant into biochar through pyrolysis offers a circular-economy strategy that may help reduce unwanted aquatic biomass while producing a value-added agricultural amendment (Jayathilake et al., 2024; Sultana et al., 2023). Although biochar has been widely studied for its beneficial effects on soil properties and plant stress responses (Jiménez-Quesada et al., 2016; Simoni et al., 2024), its role in stevia cultivation under drought conditions remains insufficiently understood. In particular, little is known about how *S. cucullata*-derived biochar affects stevia growth, antioxidant-related biochemical responses, steviol glycoside accumulation, and the expression of selected genes involved in steviol glycoside biosynthesis. It also remains unclear whether the observed plant responses are primarily driven by soil-mediated changes in water retention, pH, electrical conductivity, nutrient availability, or by more direct plant physiological responses. Addressing this gap is important for evaluating the potential of S. cucullata-derived biochar as a sustainable amendment for supporting stevia growth and metabolite production under water-limited conditions.

Therefore, this study investigated the effects of *S. cucullata*-derived biochar on *S. rebaudiana* grown under different water-holding capacities. The specific objectives were to: (1) evaluate biochar-associated changes in steviol glycoside accumulation and the expression of selected biosynthesis-related genes, and (2) characterize the physiological and antioxidant-related biochemical responses of stevia to drought stress in the presence and absence of biochar. The findings provide evidence on the potential use of *S. cucullata*-derived biochar as a sustainable soil amendment while recognizing that the underlying soil-plant and molecular mechanisms require further clarification.

## 2 Materials and methods

### 2.1 Biochar preparation and characterization

*S. cucullata* biomass was collected, washed thoroughly with tap water followed by distilled water to remove debris and contaminants, and oven-dried at 70 °C until constant weight was achieved. The dried biomass was ground into a fine powder using a mechanical grinder and passed through a 0.5 mm sieve to ensure particle uniformity. Biochar was prepared following the pyrolysis method described by Agrafioti et al. (2013) with minor modifications. Approximately 250 g of *S. cucullata* powder was placed in a stainless-steel pyrolysis reactor and heated at 300 °C for 45 min under oxygen-limited conditions. The reactor was allowed to cool to room temperature under inert atmosphere before biochar recovery. The resulting biochar was ground and sieved through a 0.5 mm mesh for homogeneity prior to soil application.

Biochar pH and electrical conductivity were measured according to the method of Singh et al. (2017) with slight modifications. Briefly, 1.0 g of dried biochar was mixed with 10 mL of deionized water and shaken at room temperature for 5 min. The suspension was then allowed to stand for 30 min. The pH was measured using a calibrated pH meter, whereas electrical conductivity was measured using a calibrated conductivity meter and reported as dS cm⁻¹ at 25 °C.

Total carbon and total nitrogen were determined using a CHN elemental analyzer. Finely ground dried biochar was weighed into tin capsules and combusted under an oxygen-rich atmosphere. The released gases were quantified by the detector system of the elemental analyzer, and the results were expressed as percentage of dry weight. The C/N ratio was calculated by dividing total carbon by total nitrogen on a mass basis:


$$\frac{C}{N}=\frac{Total\;carbon\;(\%)\;}{Total\;nitrogen\;(\%)}$$

Ash content was determined by dry combustion in a muffle furnace. Approximately 1.0 g of dried, ground biochar was placed in a pre-weighed ceramic crucible and heated at 550 °C until constant weight. After cooling in a desiccator, the crucible was weighed, and ash content was calculated as:


$$Ash\;content\;(\%)=\frac{W_{ash}}{W_{biochar}}\times 100$$

where W_ash_ is the weight of the remaining inorganic residue and W_biochar_ is the initial dry weight of biochar.

Water-holding capacity (WHC) was determined using a saturation and drainage method. Dried biochar was placed in a tube and saturated with deionized water for 24 h. After saturation, excess free water was allowed to drain for 2 h. The saturated biochar was weighed, dried at 40 °C until constant weight, and weighed again. Water-holding capacity was calculated as:


$$WHC\;(\%(=\frac{W_{saturated}-W_{dry}}{W_{dry}}\times 100$$

where W_saturated_ is the weight of water-saturated biochar after drainage and W_dry_ is the dry weight of biochar.

Scanning electron microscopy was used to qualitatively observe the surface morphology and pore structure of the biochar.

### 2.2 Experiment 1: effect of *S. cucullata*-derived biochar on steviol glycoside content and selected biosynthesis-related gene expression in Stevia

#### 2.2.1 Plant material, experimental design, and treatments

Two-month-old *S. rebaudiana* seedlings (n = 120) were obtained from a commercial nursery in Samoeng District, Chiang Mai, Thailand (18°54’26.2”N, 98°33’60.0”E). Seedlings were transplanted into 2 L pots, each pot containing 1.5 kg of agricultural soil (loam soil with 50% sand, and 15% clay) and subjected to four amendment treatments. For amended treatments, 70 g of the substrate was replaced by dried *S. cucullata*, biochar, or their mixtures according to the ratios shown in **Table 1**. The control treatment received no amendment. The experiment was conducted in a 50% shade greenhouse at Chiang Mai University (18°45’54.4”N, 98°55’50.4”E) during July 2025 (mean temperature: 27.8 °C; total rainfall: 199.1 mm) using a randomized complete block design with four replications.

**Table 1:** Soil substrate combinations with dried *Salvinia cucullata* (Asian water moss) (W) and its biochar (B) with control soil (loam soil with 50% and, 15% clay).

Treatments	Code	*S. cucullata (g)*	*S. cucullata* biochar (g)	Control (g)
1	C	0	0	70
2	W	70	0	0
3	B	0	70	0
4	W+B	35	35	0

#### 2.2.2 Identification and quantification of steviol glycosides

Steviol glycosides (SGs) were identified and quantified using a Chromaster 5000 high-performance liquid chromatography (HPLC) system (HITACHI, Tokyo, Japan) equipped with an autosampler (Chromaster 5210) and diode array detector (Chromaster 5430). Reverse-phase separation was performed on a C18 column (250 × 4.6 mm, 5 µm; COSMOSIL, Kyoto, Japan). The mobile phase consisted of solvent A (water containing 0.1% formic acid) and solvent B (acetonitrile). To optimize peak resolution for the plant matrix, a linear gradient elution program was applied over an 18 min runtime: 0–2 min, 70 % A; 2-4 min, 60 % A; 4-8 min, 20 % A; 8-9 min, 25 % A; 9-9.1 min, 95 % A; 9.1-13 min, 80 % A; 13.1-18 min, 70 % A. The flow rate was 1.0 mL/min, and the injection volume was 25 µL (Ghose et al., 2022; Simoni et al., 2024; Sunanta et al., 2024). The column oven temperature was maintained at a constant 40 °C to improve retention-time stability. Individual SGs were identified by comparing sample retention times with those of authentic standards. Quantification was performed using the external standard method by integrating peak areas and comparing them with established calibration curves. The limits of detection (LOD) and limit of quantification (LOQ) were calculated from the standard deviation of the response and the slope of the calibration curve according to the following equations:


$$LOD=\frac{3.3\sigma }{S}$$

$$LOQ=\frac{10\sigma }{S}$$

where σ is the standard deviation of the response and S is the slope of the calibration curve.

#### 2.2.3 Gene expression analysis

Leaf tissues from the 3^rd^–4^th^ node of *S. rebaudiana* plants were harvested at peak stress (week 4), immediately flash-frozen in liquid nitrogen, and stored at -80 °C following Simoni et al. (2024). Three genes involved in steviol glycoside biosynthesis were analyzed (**Supplementary Table 1**), namely *KAH*, UDP-glycosyltransferase (*UGT*74G1), and UDP-glycosyltransferase (*UGT*76G1), according to Pandey and Chikara (2015). Total RNA was extracted using the Dendrobium Total RNA/DNA Extraction and Purification Kit according to the manufacturer’s instructions. RNA concentration and purity were determined using a NanoDrop spectrophotometer. Complementary DNA (cDNA) was synthesized from 1 µg of total RNA using the reverse transcription kit (RScript cDNA Synthesis Kit (Bio-Helix, New Taipei City, Taiwan). Quantitative real-time PCR (qPCR) was conducted in a 20 µL reaction volume containing 100 ng of cDNA, 0.4 µL of each primer (10 µM), and 10 µL of 2x iTaq Universal SYBR Green Supermix (BIO-RAD, USA). Amplification was performed using a CFX Connect Real-Time PCR Detection System (BIO-RAD, USA) under the following conditions: initial denaturation at 95 °C for 30 s, followed by 40 cycles of 95 °C for 15 s and 60 °C for 30 s. Melt-curve analysis was performed to confirm amplification specificity. Relative gene expression was calculated using the 2^−ΔΔCt^ method (Livak and Schmittgen, 2001), with 18S RNA used as the reference gene.

### 2.3 Experiment 2: Role of *S. cucullata* Biochar on Physiological and Biochemical Responses of Stevia to Drought Stress

#### 2.3.1 Biochar amendments and drought-stress experiment

Experiment 2, six soil amendment treatments were established (**Table 2**). The experiment was arranged in a randomized complete block design with four replications, and all cultivation conditions were identical to those described in Experiment 1.

**Table 2:** Soil substrate combinations with dried *Salvinia cucullata* (Asian water moss) (W) and its biochar (B) with control soil (loam soil with 50% and, 15% clay).

Treatments	Code	*S. cucullata (g)*	*S. cucullata* biochar (g)	Control (g)
1	C	0	0	70
2	W	70	0	0
3	B	0	70	0
4	WB (1:1)	35	35	0
5	WB (3:1)	52.5	17.5	0
6	WB (1:3)	17.5	52.5	0

Drought stress was imposed by regulating soil water-holding capacity (WHC), calculated as follows:


$$\text{WHC }(\%)=(\frac{\text{W}1-\text{W}2}{\text{W}1})\times 100$$

where W1 is the dry soil weight and W2 is the soil weight after 24 h drainage.

Four WHC levels were maintained: 100% WHC (control), 85% WHC, 65% WHC, and 45% WHC, following Karimi et al. (2015). Plant height, new shoot number, chlorophyll content index (SPAD-502, Konica Minolta), leaf area index (LAI), total biomass, and root biomass were recorded at week four.

#### 2.3.2 Biochemical analyses

Fresh leaf samples were used immediately for electrolyte leakage analysis (Valentovic et al., 2006), whereas the remaining tissue was oven-dried at 40 °C for 12 h and ground into a fine powder. Then, dried powder (0.1 g) was mixed with 1 mL of 80% ethanol, sonicated for 30 min (Ultrasonicator, Beijing Ultrasonic, China), and centrifuged at 10,000 xg for 10 min at 25 °C (1-16K refrigerated Centrifuge, Sigma, Germany). The supernatant was collected for subsequent biochemical assays.

##### 2.3.2.1 Total phenolic and total flavonoid content

Total phenolic content was quantified using the Folin-Ciocalteu method modified from Charoimek et al. (2024). Extract (30 µL) was mixed with 60 µL Folin-Ciocalteu reagent and 210 µL of 6% sodium bicarbonate solution, then incubated in darkness for 2 h at room temperature. Absorbance was measured at 725 nm using a microplate reader (SpectraMax iD3, USA). Results were expressed as mg gallic acid equivalents (GAE) per gram dry weight based on a standard curve (10–200 mg/mL).

Total flavonoids were determined according to Charoimek et al. (2024). Extract (25 µL) was mixed with 125 µL distilled water and 7.5 µL of 5% NaNO₂, incubated for 5 min, followed by addition of 15 µL of 10% AlCl₃·6H₂O for 6 mins. Finally, 50 µL of 1 M NaOH and 27.5 µL distilled water were added. Absorbance at 510 nm was measured and results expressed as mg catechin equivalents (CE) per gram dry weight using a standard curve (30–300 mg/mL).

##### 2.3.2.2 Antioxidant activity

DPPH radical scavenging activity was assessed following Charoimek et al. (2024). Extract (25 µL) was mixed with 250 µL of 0.2 mM DPPH solution and incubated in darkness for 30 min. Absorbance was measured at 510 nm, and scavenging activity calculated as follows:


$$\text{DPPH scavenging activity }(\%)=\frac{A_{\text{control}}-A_{\text{sample}}}{A_{\text{control}}}\times 100$$

ABTS radical scavenging activity was determined by preparing an ABTS⁺ radical cation solution (7.0 mM ABTS with 2.45 mM potassium persulfate, incubated for 12–16 h in darkness). The working solution was diluted to an absorbance of 0.70 ± 0.02 at 734 nm. Extract (10 µL) was mixed with 200 µL of ABTS working solution, incubated for 30 min, and absorbance measured at 734 nm. Scavenging activity was calculated using the same equation described above.

##### 2.3.2.3 Analysis of Phenolic Compounds

Phenolic compounds were quantified using a Shimadzu HPLC system (SPD-10AV, LC-10AD, SIL-10AD, Japan) with UV-Vis detection, modified from Sangta et al. (2021). Separation was performed on a C18 column using gradient elution with mobile phases consisting of (A) acetonitrile/formic acid/water (94.9:0.1:5, v/v/v) and (B) 0.1% formic acid in water. The gradient program was as follows: 80% B initially, decreased to 30% B over 3 min and held for 1 min, increased to 55% B for 1 min, decreased to 10% B, increased to 60% B over 3 min and held for 3 min, and returned to 80% B for 3 min. Flow rate was 1.0 mL/min, the injection volume was 10 µL and the total run time was 16 min. Gallic acid and quercetin standards (0–1000 ppm in HPLC-grade methanol) were used for calibration.

### 2.4 Statistical analysis

The experiment was arranged as a randomized complete block design with four blocks. Each treatment combination included four plants per block. For biochemical and qPCR analyses, four independent biological replicates were used. Two-way analysis of variance (Two-way ANOVA) was used to evaluate the main effects of soil amendment treatment, WHC level, and their interaction on measured parameters. Tukey’s honestly significant difference test was applied for *post-hoc* comparisons among treatment means. All statistical analyses were performed using R software (version 4.3.3), and data visualisation was conducted using the ggplot2 package.

## 3 Results

### 3.1 Biochar characterization

The physicochemical properties of the *S. cucullata*-derived biochar are presented in **Table 3**. The biochar was alkaline (pH of 8.83 ± 0.09), indicating that it may influence substrate pH when applied as a soil amendment. Its electrical conductivity was 6.03 ± 0.55 dS/m, suggesting the presence of soluble ionic constituents. The material also had very low moisture content (0.13 ± 0.03%) and relatively high ash content (40.57 ± 0.80%), indicating that a substantial proportion of inorganic fraction remained after pyrolysis. This high ash content may reflect the aquatic origin of *S. cucullata*, and its capacity to accumulate mineral components from the surrounding environment. Volatile matter accounted for 40.03 ± 1.10%, indicating that part of the organic fraction remained in a thermally labile. Elemental analysis showed that the biochar contained 19.27 ± 1.86% total carbon and 0.96 ± 0.30% total nitrogen, resulting in a C/N ratio of 20.99 ± 5.07. The water-holding capacity of the biochar was 232.50 ± 5.85%, indicating that it retained more than twice its dry weight in water under the measurement conditions. This property suggests potential to contribute to substrate water retention, although the actual effect on soil water availability would depend on soil type, application rate, and cultivation conditions.

**Table 3:** The physicochemical properties of the *S. cucullata*-derived biochar.

Measurments	Value
pH	8.83±0.09
Electrical conductivity	6.03±0.55
Moisture (%)	0.13±0.03
Ash (%)	40.57±0.80
Volatile Matter (%)	40.03±1.10
Total carbon (%)	19.27±1.86
Total nitrogen (%)	0.96±0.30
C/N ratio	20.99±5.07
WHC(%)	232.50±5.85

Values are mean ± SD.

**Figure 1:**
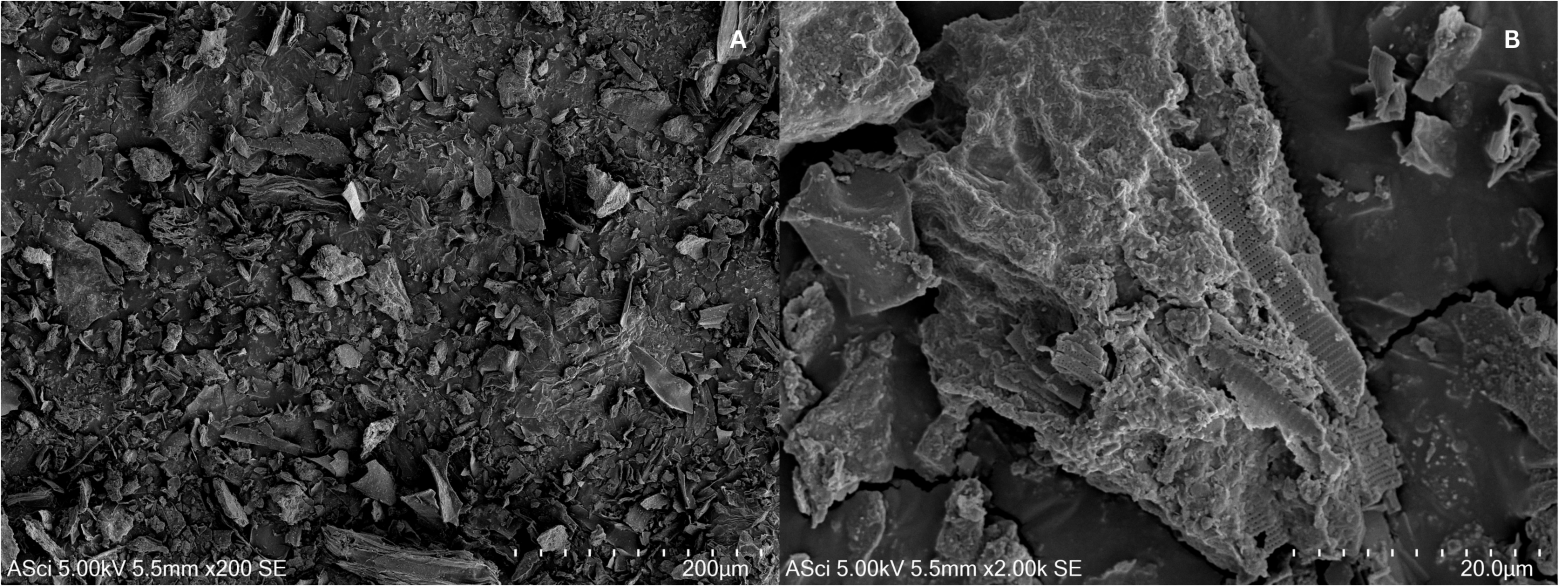
SEM images of *S. cucullata*-derived biochar showing a rough and heterogeneous surface structure at magnifications of 200× **(A)** and 2000× **(B)**.

SEM analysis showed that the *S. cucullata*-derived biochar had a rough, heterogeneous, and fragmented surface structure, with visible cracks, irregular particles, and pore spaces formed during pyrolysis (**Figure 1**). These features are consistent with the thermal decomposition and partial collapse of plant-derived biomass. An elongated structure with ordered micron-scale perforations was also observed on the biochar surface. Based on morphology, this structure resembled a diatom frustule, or silica-based valve-like shell, embedded within the biochar matrix. This observation is consistent with the aquatic origin of *S. cucullata*, which was collected from surface-flowing freshwater environments in Thailand where benthic diatoms may attach to submerged or floating plant surfaces (Leelahakriengkrai and Kunpradid, 2018). If confirmed, the persistence of this structure after pyrolysis may be related to its silica-rich composition. Such siliceous residues could contribute to the mineral fraction and surface complexity of the biochar and may also be consistent with the relatively high ash content observed in **Table 1**. However, SEM morphology alone cannot confirm its elemental composition. Further analysis, such as SEM-EDS, would be required to verify whether the observed structure is silica-rich and to confirm its identification as a diatom-derived feature.

### 3.2 Experiment 1

#### 3.2.1 *S. cucullata*-derived biochar-mediated changes in steviol glycoside contents

**Figure 2:**
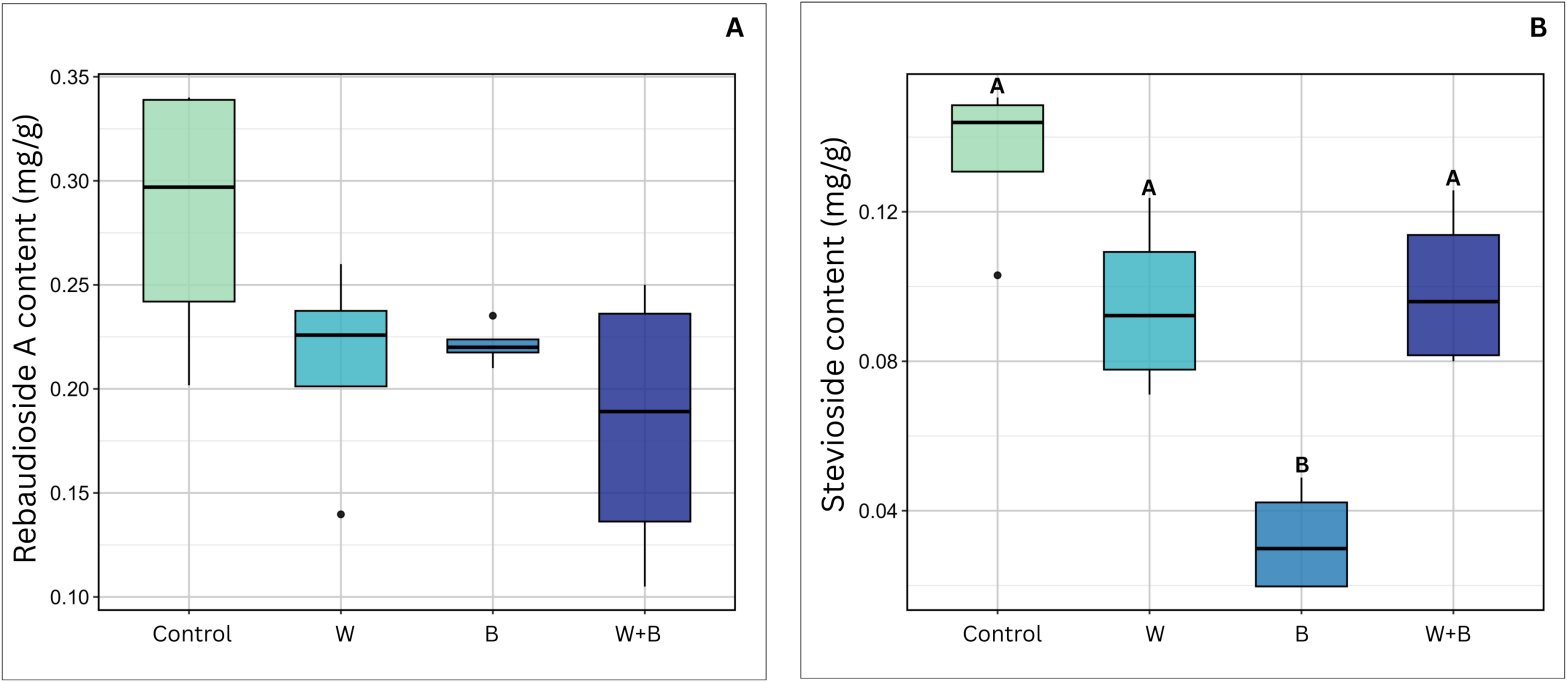
Rebaudioside A and stevioside content in stevia under different soil treatments. Control, Soil without amendment; W, Soil amended with water moss; B, Soil amended with *Salvinia cucullata*-derived biochar; W+B, Soil amended with both water moss and *(S) cucullata*-derived biochar. Different letters indicate statistically significant differences among treatments according to Tukey’s multiple comparison test (p < 0.05).

The concentrations of the two major steviol glycosides, stevioside and rebaudioside A, varied among soil amendment treatments (**Figure 2**). The LOD values for stevioside and rebaudioside A were 0.02 and 0.01 mg/g, respectively, while the corresponding LOQ values were 0.05 and 0.02 mg/g. The control treatment (C) showed the highest concentrations of both compounds, with stevioside and rebaudioside A levels of 0.14 ± 0.02 mg/g and 0.28 ± 0.07 mg/g, respectively. The water moss treatment (W) was associated with slightly lower concentrations, with 0.09 ± 0.02 mg/g stevioside and 0.21 ± 0.05 mg/g rebaudioside A. In plants grown with *S. cucullata*-derived biochar (B), stevioside concentration decreased substantially to 0.03 ± 0.01 mg/g, whereas rebaudioside A remained within a range comparable to the other amendment treatments (0.22 ± 0.01 mg/g). The combined water moss and biochar treatment (W+B) produced intermediate values, with stevioside and rebaudioside A recorded at 0.10 ± 0.02 mg/g and 0.18 ± 0.07 mg/g, respectively. Tukey’s multiple comparison test indicated that only the stevioside concentration in the B treatment differed significantly from the other treatments (*p* < 0.05). By contrast, rebaudioside A did not vary significantly among treatments, suggesting that individual steviol glycosides responded differently to amendment conditions.

#### 3.2.2 *S. cucullata*- derived biochar-mediated changes in selected steviol glycoside biosynthesis-related gene expression in Stevia

**Figure 3:**
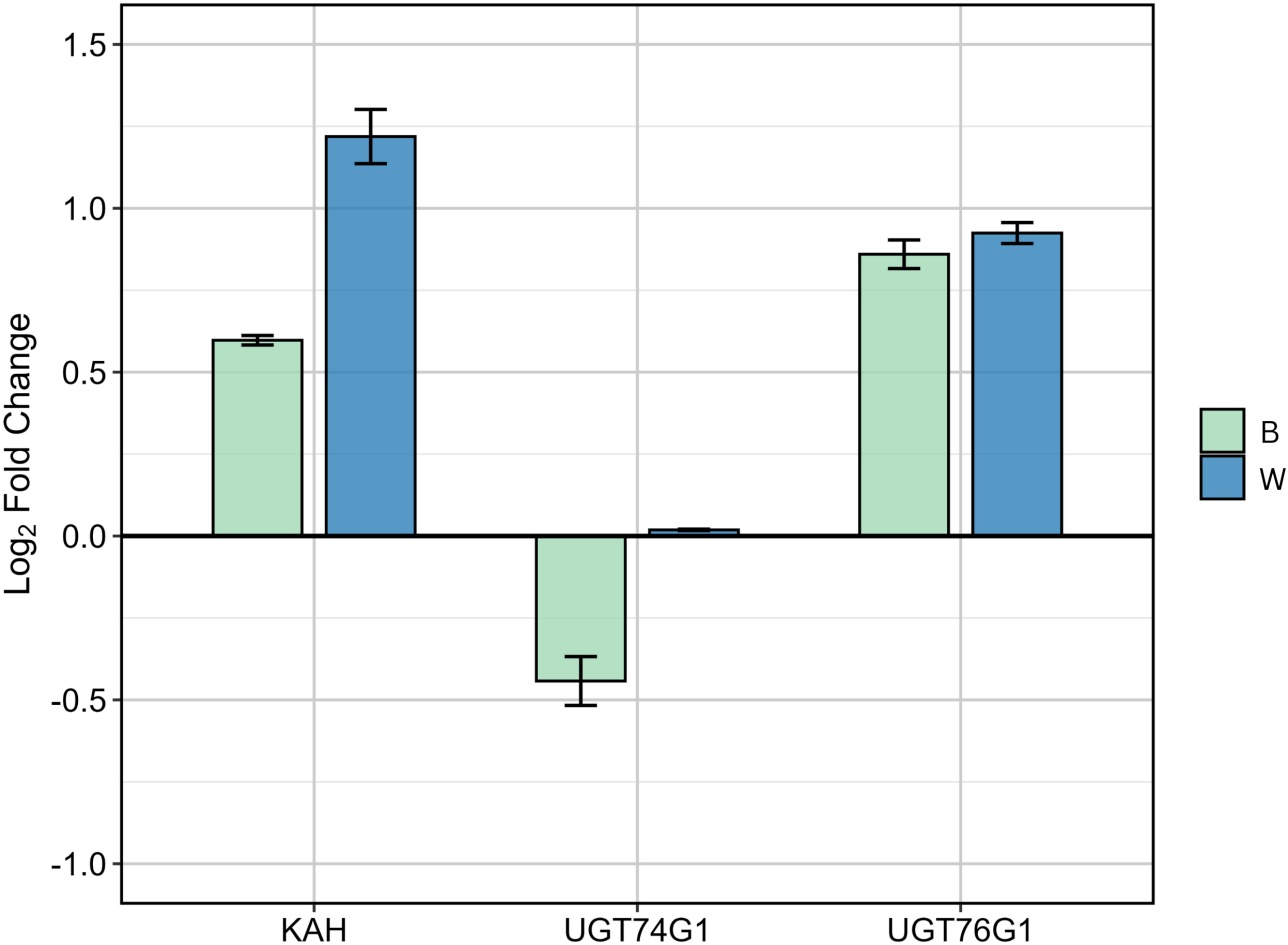
The gene expression levels of steviol glycoside under different soil treatments.

The qPCR analysis indicated gene-specific transcriptional responses within selected steviol glycoside biosynthetic related genes in *S. rebaudiana* under different soil amendment treatments. Relative transcript abundance was calculated using the 2^-ΔΔCq^ method, with the control treatment used as the calibrator and assigned a fold-change value of 1 (**Figure 3**). The expression of KAH which encodes kaurenoic acid hydroxylase and contributes to the conversion of ent-kaurenoic acid toward steviol formation, was upregulated under both amendment treatments. The W treatment exhibited the highest KAH transcript abundance, with a fold change of 2.25 corresponding to a Log_2_FC of 1.17, whereas the B treatment showed a more moderate 1.49-fold increase and a Log_2_FC of 0.58. This pattern suggests that water moss amendment was more strongly associated with increased KAH transcript abundance than biochar alone. UGT74G1, which encodes a UDP-glycosyltransferase involved in steviol glycoside formation, showed limited variation across treatments. The W treatment produced an expression level nearly equivalent to the control, with a 1.01-fold change and a Log_2_FC of 0.02. The B treatment showed a modest reduction in UGT74G1 transcription abundance, with a 0.81-fold change and a Log_2_FC of −0.31. In contrast, UGT76G1, which is associated with the conversion of stevioside to rebaudioside A, was upregulated under both amendment treatments. The W treatment showed a 1.97-fold increase, corresponding to a Log_2_FC of 0.98, while B treatment produced a comparable response, with a 1.86-fold increase and a Log2FC of 0.89. These findings indicate that water moss and biochar amendments were associated with different expression patterns among selected steviol glycoside biosynthesis-related genes; however, these targeted qPCR data should not be interpreted as evidence of pathway-wide regulation.

### 3.3 Experiment 2

#### 3.3.1 Physical and morphological study

**Figure 4:**
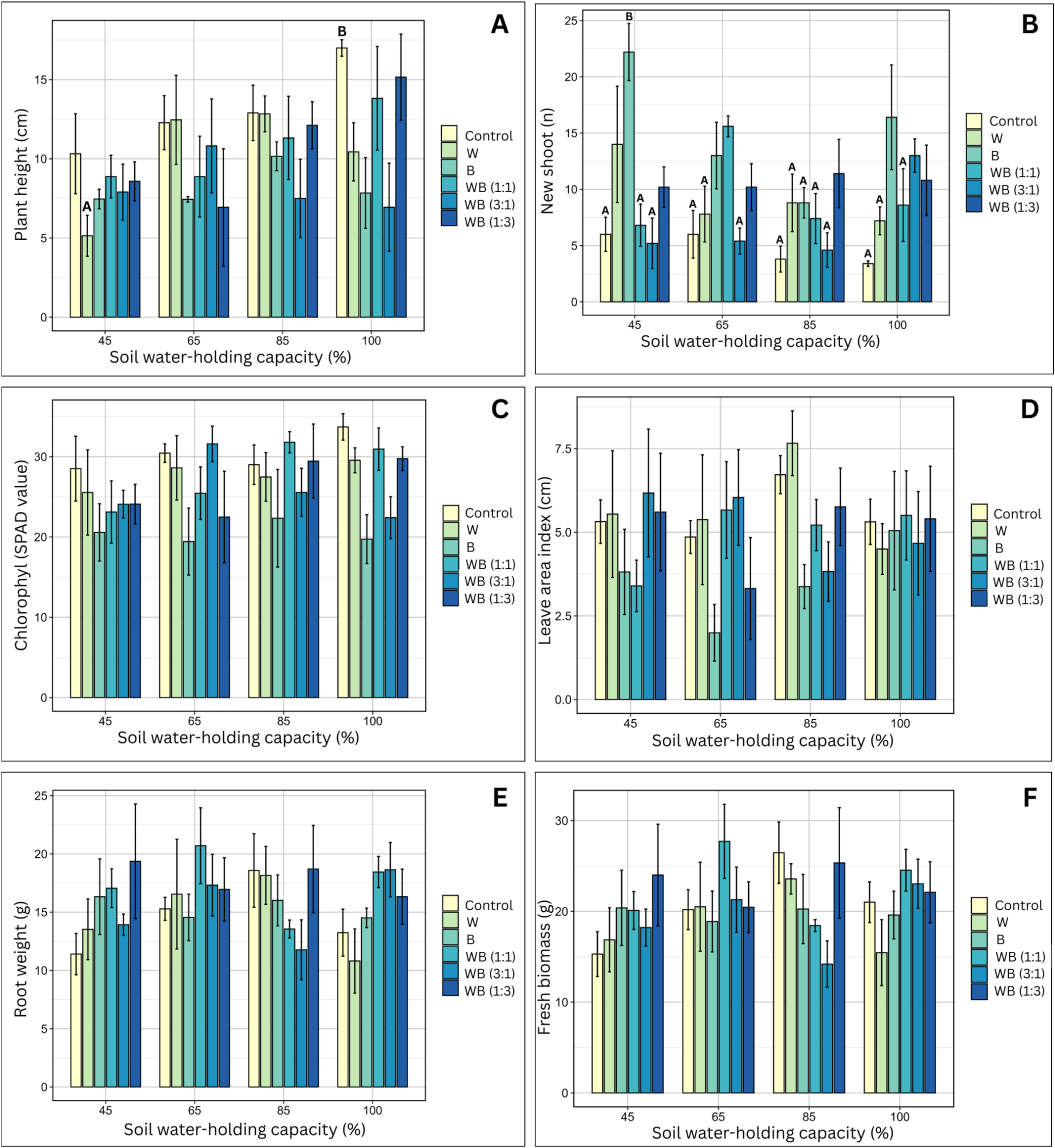
Physiological and morphological parameters of *Stevia rebaudiana* across all experimental treatments at week four. The plots illustrate **(a)** plant height (cm), **(b)** number of new shoots, **(c)** chlorophyll content (SPAD), **(d)** leaf area index (LAI), **(e)** total biomass, and **(f)** root weight. Data are presented as means of the three biological replicates (n=5) for each combination of Water Holding Capacity (100%, 85%, 65%, and 45% WHC) and soil amendment treatment with their standard error (SE). Different letters indicate statistically significant differences among treatments according to Tukey’s multiple comparison test (p < 0.05).

As shown in **Figure 4** and **Supplementary Table 1**, WHC level and soil amendment treatment influenced selected growth traits of *S. rebaudiana*, although the responses were not consistent across all measured parameters. Plant height was highest in the control soil at 100% WHC (17.13 ± 1.30 cm), and lowest in the W treatment at 45% WHC (5.14 ± 2.89 cm) (**Figure 4A**). However, significant differences in plant height were limited to specific treatment combination, suggesting that vertical growth was only partly influenced by the imposed conditions. The number of new shoots showed a clearer treatment-related pattern. The B treatment at 45% WHC had the highest mean shoot number per plant (22.20 ± 5.67) (**Figure 4B**), and biochar-containing treatment, particularly B and WB (1:3), were generally associated with greater shoot production than non-biochar treatments. The chlorophyll content index, measured using SPAD, showed treatment-related variation in the two-way ANOVA (**Figure 4C**). However, this response did not correspond to consistent changes in other growth indicators. Leaf area index, total biomass, and root weight did not differ significantly among soil treatments or WHC levels (**Figures 4D–F**). These results indicate that the amendments influenced specific morphological and chlorophyll-related traits but did not produce a consistent increase in whole-plant growth.

The two-way ANOVA results further supported this interpretation (**Table 4**). WHC significantly affected plant height, whereas soil amendment treatment significantly affected plant height (F-value 2.92, *p*-value 0.02), number of new shoots (F-value 8.03, *p*-value < 0.001), and SPAD values (F-value 3.79, *p*-value < 0.001). However, soil amendment treatment had no significant effect on LAI, total biomass, or root weight. No significant WHC × Treatment interaction was observed for any measured variable, indicating that treatment effects were not strongly dependent on WHC level under the conditions tested. Therefore, these findings do not provide sufficient evidence to conclude that biochar enhanced drought tolerance. Rather, the results show that biochar amendment was associated with specific changes in growth architecture, particularly shoot production, while most whole-plant growth indicators remained statistically unchanged.

**Table 4:** Two-way analysis of variance (ANOVA) results for *Stevia rebaudiana* seedlings.

Source of variation	Dependent variable	Sum of squares	df	Mean square	F-value	p-value	Sig.
WHC (%)	Plant Height (cm)	253.6	3	84.55	3.705	0.0143	*
	New Shoots (n)	174.9	3	58.30	1.927	0.1304	ns
	Chlorophyll (SPAD)	222.0	3	73.84	1.273	0.2881	ns
	LAI (cm)	11.9	3	3.97	0.485	0.693	ns
	Biomass (g)	107.0	3	35.83	0.627	0.600	ns
	Root (g)	53.0	3	17.64	0.505	0.680	ns
Treatment	Plant Height (cm)	332.6	5	66.52	2.915	0.0171	*
	New Shoots (n)	1215.3	5	243.07	8.034	0.0000	***
	Chlorophyll (SPAD)	1099.0	5	219.78	3.788	0.004	**
	LAI (cm)	60.3	5	12.05	1.470	0.207	ns
	Biomass (g)	297.0	5	59.38	1.039	0.334	ns
	Root (g)	192.0	5	38.34	1.098	0.367	ns
WHC × Treatment	Plant Height (cm)	410.2	15	27.34	1.198	0.2866	ns
	New Shoots (n)	1060.1	15	70.68	1.667	0.071	ns
	Chlorophyll (SPAD)	644.0	15	42.91	0.740	0.7387	ns
	LAI (cm)	104.0	15	6.93	0.846	0.625	ns
	Biomass (g)	976.0	15	65.10	1.139	0.334	ns
	Root (g)	575.0	15	38.30	1.096	0.370	ns
Residuals	Plant Height (cm)	2191.0	96	22.82	–	–	–
	New Shoots (n)	2904.4	96	30.25	–	–	–
	Chlorophyll (SPAD)	5569.0	96	58.01	–	–	–
	LAI (cm)	786.9	96	8.19	–	–	–
	Biomass (g)	5489.0	96	57.17	–	–	–
	Root (g)	3354.0	96	34.93	–	–	–

Analysis of Variance; *p <.05, **p <.01, ***p <.001, ns=nonsignificant. The table evaluates the effects of Water Holding Capacity (WHC: 100%, 85%, 65%, 45%), *Salvinia cucullata*-derived biochar treatment [C, W, B, WB (1:1), WB (1:3), WB (3:1)], and their interaction (WHC × Treatment) at week four of growth. Dependent variables include Plant Height, Number of New Shoots, Total Biomass, Root Weight.

#### 3.3.2 Physicochemical and chemical study

**Figure 5:**
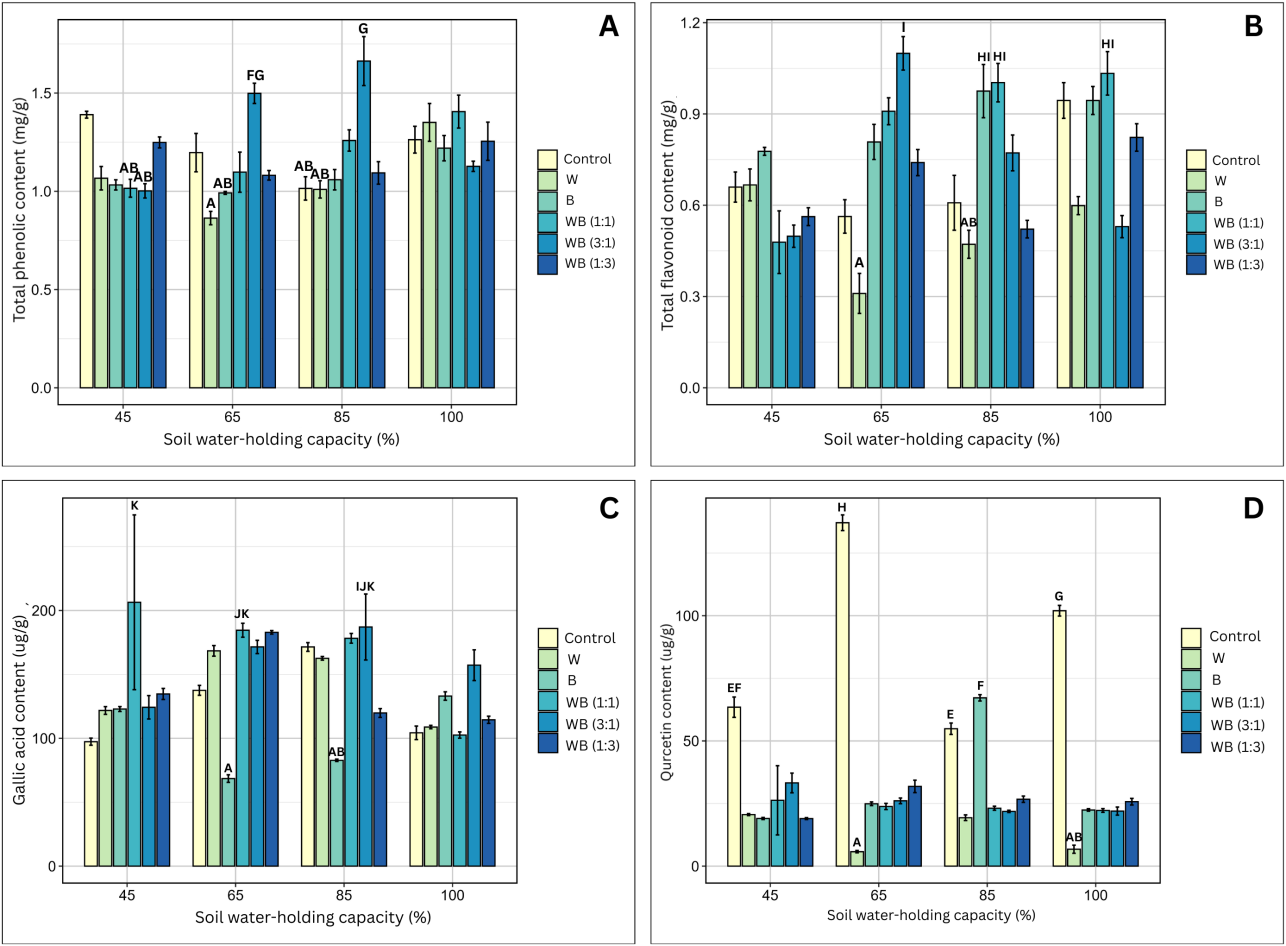
Chemical parameters of *Stevia rebaudiana* across all experimental treatments at week four. The plots illustrate **(a)** total phenolic content (TPC), **(b)** total flavonoid content (TFC), **(c)** gallic acid, **(d)** quercetin. Data are represented as means with standard error of the three biological replicates (n=5) for each combination of water holding capacity (100%, 85%, 65%, and 45% WHC) and soil amendment treatment. Different letters indicate statistically significant differences among treatments according to Tukey’s multiple comparison test (p < 0.05).

As shown in **Figure 5**, secondary metabolite accumulation varied according to WHC level and amendment treatment. Total phenolic content (TPC) was highest in the WB (3:1) treatment at 85% WHC, reaching 1.66 ± 0.43 mgGAE/g, followed by the same treatment at 65% WHC (1.50 ± 0.02). A similar trend was observed for total flavonoid content (TFC). At 65% WHC, the WB (3:1) treatment showed the highest TFC value (1.10 ± 0.19 mgCAE/g), which was approximately twofold higher than that of the control (0.56 ± 0.19 mgCAE/g) at the same WHC level. Under lower water availability at 45% WHC, the B treatment maintained relatively high flavonoid content (0.78 ± 0.04 mgCAE/g), suggesting that biochar-containing amendments may be associated with enhanced flavonoid accumulation under water-limited conditions. The accumulation of individual phenolic compounds also differed among treatments. Gallic acid was relatively high in the WB (1:1) treatment, with concentrations of 206.42 ± 96.65 μg/g at 45% WHC and 184.56 ± 10.00 μg/g at 65% WHC. Conversely, quercetin concentration was consistently higher in the control treatment than in the amended treatments across WHC levels. These results indicate that amendment treatments may influence the relative accumulation of specific phenolic compounds, although the present data do not confirm changes in phenylpropanoid pathway regulation.

**Figure 6:**
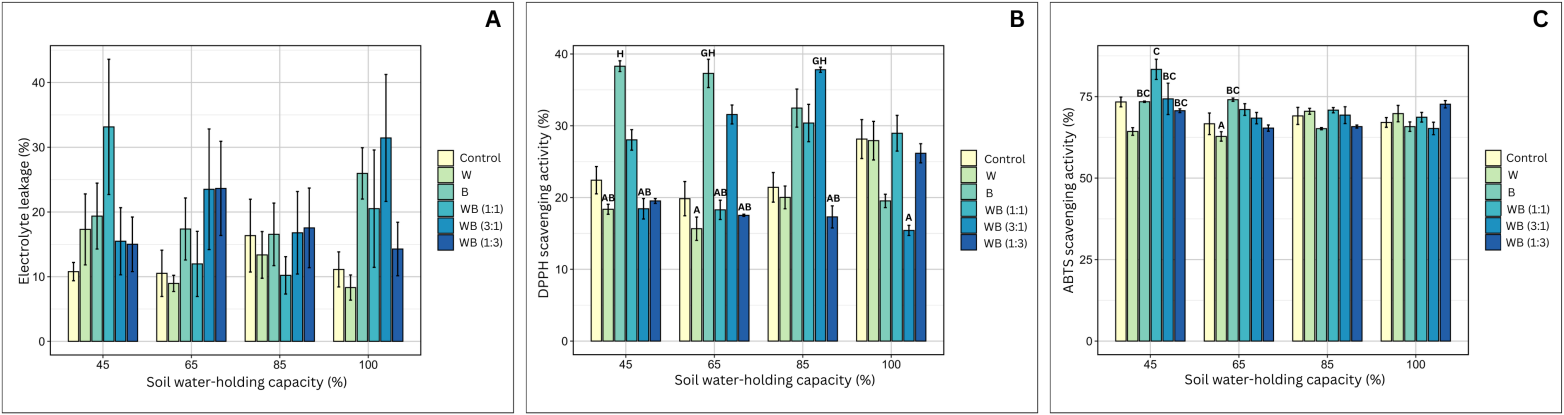
Electrolyte leakage and antioxidant activity of *Stevia rebaudiana* across all experimental treatments at week four. The plots illustrate **(a)** electrolyte leakage (EL), **(b)** DPPH radical scavenging activity, **(c)** ABTS radical scavenging activity. Data are represented as means with standard error of the three biological replicates (n=5) for each combination of water holding capacity (100%, 85%, 65%, and 45%) and soil amendment treatment. Different letters indicate statistically significant differences among treatments according to Tukey’s multiple comparison test (p < 0.05).

Antioxidant activity also varied among treatments and WHC levels (**Figure 6**). ABTS radical scavenging activity ranged from 63% to 83%, indicating the overall antioxidant capacity was broadly maintained across the experimental conditions. DPPH scavenging activity was highest in the B treatment at 45% WHC (38.20%), followed by the B treatment at 65% WHC. In contrast, the W treatment demonstrated lower DPPH activity under reduced WHC. Electrolyte leakage (EL), used as an indicator of membrane integrity, did not differ significantly among treatments, indicating that the observed biochemical variation was not accompanied by statistically detectable changes in membrane damage.

The two-way ANOVA results supported the influence of both WHC level and soil amendment treatment on biochemical responses (**Table 5**). WHC significantly affected TPC, TFC, DPPH, and ABTS, indicating that water availability contributed to variation in antioxidant-related traits. Soil amendment treatment also significantly affected TPC (F = 7.013, *p* < 0.001), TFC (F = 13.50, *p* < 0.001), ABTS (F = 10.050, *p* < 0.001), as well as DPPH (F = 3.766, *p* = 0.0113). In contrast, EL was not significantly affected by treatment (*p* = 0.6382), despite apparent variation in the descriptive data.

**Table 5:** Univariate analysis of variance (ANOVA) results following a Multivariate Analysis of Covariance (MANCOVA) for *Stevia rebaudiana* seedlings.

Source of variation	Dependent variable	Sum of squares	df	Mean square	F-value	p-value	Sig.
WHC (%)	Electrolyte Leakage (%)	280.7	3	93.5	0.567	0.6382	ns
	TPC (mg/g)	1.034	3	0.3446	7.013	0.0001	***
	TFC (mg/g)	1.555	3	0.5184	13.50	0.0000	***
	DPPH (%)	405.0	3	135.0	3.766	0.0113	*
	ABTS (%)	1362.0	3	454.0	10.050	0.0000	***
Treatment	Electrolyte Leakage (%)	1668.1	5	333.6	2.022	0.0823	ns
	TPC (mg/g)	2.122	5	0.4244	8.637	0.0000	***
	TFC (mg/g)	4.335	5	0.8670	22.58	0.0000	***
	DPPH (%)	4684.0	5	936.7	26.13	0.0000	***
	ABTS (%)	1147.0	5	229.4	5.079	0.0000	***
WHC × Treatment	Electrolyte Leakage (%)	3095.5	15	206.4	1.251	0.2489	ns
	TPC (mg/g)	6.564	15	0.4376	8.905	0.0000	***
	TFC (mg/g)	6.640	15	0.4427	11.53	0.0000	***
	DPPH (%)	9534.0	15	635.6	17.735	0.0000	***
	ABTS (%)	2927.0	15	195.2	4.321	0.0000	***
Residuals	Electrolyte Leakage (%)	15836.0	96	165.0	–	–	–
	TPC (mg/g)	12.974	96	0.0491	–	–	–
	TFC (mg/g)	10.136	96	0.0384	–	–	–
	DPPH (%)	9461.0	96	35.8	–	–	–
	ABTS (%)	11925.0	96	44.2	–	–	–

Analysis of Variance; *p <.05, **p <.01, ***p <.001, ns=nonsignificant. The table evaluates the effects of Water Holding Capacity (WHC: 100%, 85%, 65%, 45%), *Salvinia cucullata*-derived biochar treatment (C, W, B, WB (1:1), WB (1:3), WB (3:1)), and their interaction (WHC × Treatment) at week four of growth. Dependent variables include Electrolyte Leakage (%), Total phenolic (TPC) and flavonoid (TFC) contents, antioxidant potential as determined by DPPH and ABTS assays.

When considered together with the growth data, these biochemical findings suggest that water moss and biochar amendments were associated with selective changes in secondary metabolite accumulation and antioxidant activity rather than consistent improvements in whole-plant growth. Biochar-containing treatments, particularly B and WB combinations, were linked to increased shoot production and higher levels of selected antioxidant-related compounds under some WHC conditions.

## 4 Discussion

The *S. cucullata*-derived biochar was characterized by alkaline pH, relatively high electrical conductivity, high ash content, porous and heterogeneous surface morphology, and strong water-holding capacity. These properties suggest that the biochar may have altered substrate conditions by influencing pH, soluble ion availability, mineral input, and water retention. Such effects are consistent with previous reports showing that biochar properties and soil responses depend strongly on feedstock type, pyrolysis conditions, ash content, porosity, and application rate (Adhikari et al., 2023; Zhu et al., 2025). Accordingly, the observed plant responses should be interpreted primarily as amendment-associated responses rather than as evidence of a direct biological elicitor effect.

Steviol glycoside biosynthesis in *Stevia rebaudiana* involves a complex sequence of diterpenoid biosynthetic reactions followed by multiple glycosylation steps. These processes include upstream MEP pathway genes, diterpene biosynthesis genes, cytochrome P450-dependent hydroxylation, and several UDP-glycosyltransferases, including UGT74G1 and UGT76G1 (Kumar et al., 2012; Libik-Konieczny et al., 2021). Therefore, the present findings should be interpreted within the limited scope of the three targeted genes and two quantified glycosides examined in this study. Biochar treatment was associated with a marked reduction in stevioside concentration, whereas rebaudioside A remained relatively stable across treatments. This pattern suggests that individual steviol glycosides may respond differently to amendment conditions. The qPCR results provide some molecular context for this response. The KAH which is involved in the conversion of ent-kaurenoic acid toward steviol formation (Kumar et al., 2012), was upregulated in both amendment treatments, particularly in the water moss treatment. However, increased KAH expression did not correspond to higher stevioside accumulation. UGT74G1, which is a UDP-glycosyltransferase responsible for adding glucose molecules to the steviol backbone leading to stevioside formation (Behroozi et al., 2017), remained almost unchanged in the W treatment and was modestly reduced under B amendment. This pattern is consistent with the absence of increased stevioside accumulation, particularly in the B treatment. In contrast, UGT76G1, which is associated with the conversion of stevioside to rebaudioside A (Behroozi et al., 2017), was upregulated under both amendment treatments. This may help explain the relative stability of rebaudioside A. Nevertheless, rebaudioside A accumulation cannot be attributed solely to UGT76G1 expression, because metabolite levels also depend on enzyme activity, precursor availability, substrate competition, compartmentalization, and turnover (Shih and Morgan, 2020).

The morphological results from Experiment 2 indicate that water availability and soil amendment influenced selected growth traits of *S. rebaudiana*, but these effects were not expressed as a consistent improvement in whole-plant growth. WHC significantly affected plant height, whereas most other growth-related variables, including leaf area index, total biomass, and root weight, showed no significant response to WHC alone. Soil amendment treatment significantly affected plant height, number of new shoots, and SPAD value, suggesting that the growing medium influenced specific aspects of plant morphology and chlorophyll-related status rather than overall biomass accumulation. Biochar-containing treatments were associated with increased shoot production, particularly under some water-limited conditions. The B treatment at 45% WHC showed relatively high lateral shoot formation, whereas vertical growth did not increase consistently. This pattern may indicate a treatment-associated change in shoot architecture rather than a general enhancement of plant growth. However, increased shoot number alone cannot be interpreted as evidence of improved drought resilience. Ming et al. (2025) have suggested that biochar can improve plant performance under water-limited conditions by modifying soil water retention, nutrient availability, and plant physiological responses. However, these effects vary substantially depending on biochar type, soil texture, application rate, and crop species (Wei et al., 2023; Zhang et al., 2024). Biochar has also been reported to increase soil water retention capacity, particularly in coarse-textured soils, although improved soil water retention does not necessarily translate into improved drought tolerance or biomass production (Ndede et al., 2022). The physicochemical properties of the *S. cucullata*-derived biochar therefore provide plausible explanations for the treatment-associated effects observed in this study.

The biochemical data suggest that the amendments were associated with selective changes in antioxidant-related metabolites. Biochar and water moss-biochar combinations increased total phenolic content, total flavonoid content, gallic acid concentration, and DPPH radical scavenging activity under some WHC conditions. These responses are consistent with the broader role of phenolic compounds and flavonoids as non-enzymatic antioxidants involved in plant responses to abiotic stress (Kumar et al., 2012). Moreover, Ahmad et al. (2020) similarly reported that water-deficit or PEG-induced stress in *S. rebaudiana* was associated with changes in growth performance, steviol glycosides, total phenolics, total flavonoids, and antioxidant activity. Although biochar-associated changes in antioxidant-related gene expression and defence-related metabolites have been reported in other plant systems, including wheat, tomato, and tobacco (Fallah et al., 2023) (Yadav et al., 2020) (Chen et al., 2025), direct evidence for similar molecular responses in *S. rebaudiana* remains limited. Electrolyte leakage did not differ significantly among treatments, despite apparent variation in the descriptive data. This result suggests that the observed changes in phenolic and flavonoid accumulation were not accompanied by statistically detectable differences in membrane injury, although electrolyte leakage is widely used as an indicator of cell membrane stability under water stress (Bajji et al., 2002).

Although the present study demonstrates that *S. cucullata-*derived biochar is associated with changes in selected physiological, biochemical, and gene-expression traits, the underlying mechanism remains unresolved. Because biochar can modify substrate pH, electrical conductivity, nutrient availability, and water retention, the observed plant responses cannot be attributed specifically to biological elicitation. Furthermore, the targeted analysis of three genes and two steviol glycosides does not allow inference of pathway-level metabolic flux. Future studies integrating soil chemistry, phytohormone profiling, ROS-related markers, antioxidant enzyme activity, transcriptomics, metabolomics, and isotope-based flux analysis are required to clarify the mechanisms underlying these amendment-associated responses.

## 5 Conclusion

This study shows that *Salvinia cucullata*-derived biochar can influence selected growth responses, antioxidant-related biochemical traits, and steviol glycoside biosynthesis-related gene expression in *Stevia rebaudiana* under different water-holding capacity conditions. Biochar-treated plants did not show a consistent increase in overall biomass or leaf area; instead, the main morphological response was reflected in altered growth architecture, particularly increased lateral shoot production under some water-limited conditions. Biochemically, biochar application was associated with higher total flavonoid content and gallic acid accumulation, and DPPH radical scavenging activity under certain water regimes. However, electrolyte leakage did not differ significantly among treatments, indicating that these biochemical changes were not accompanied by clear evidence of reduced membrane injury. At the molecular level, biochar treatment was associated with increased KAH and UGT76G1 transcript abundance and slightly reduced UGT74G1 expression. These expression patterns were consistent with reduced stevioside concentration and relatively stable rebaudioside A level under biochar treatment. In this sense, *S. cucullata*-derived biochar may serve as a potential sustainable soil amendment for modulating selected drought-associated physiological adjustments and secondary metabolite profiles in *S. rebaudiana*. Future studies should incorporate post-amendment soil properties, stress-related signaling markers, enzyme activities, and broader transcriptomic or metabolomicanalyses to clarify the underlying mechanisms.

## Data Availability

The original contributions presented in the study are included in the article/**Supplementary Material**. Further inquiries can be directed to the corresponding authors.

## Supplementary material

Supplementary Table 1 Primer sequences of genes involved in steviol glycoside biosynthesis.

Supplementary Table 2 Physiological parameters at week 4 of *Stevia rebaudiana* plants grown under drought stress conditions.

Supplementary Table 3 Comprehensive Biochemical and Antioxidant Profiles of at week 4 of *Stevia rebaudiana* plants grown under drought stress conditions.
