# Supplementary material for: Invasive Asian water moss (Salvinia cucullata) biochar modulates selected steviol glycoside biosynthesis-related gene expression and drought-associated physiological responses in Stevia
Source: Front Plant Sci. 2026 Jul 10;17:1859834. doi: 10.3389/fpls.2026.1859834 (PMC13397357; doi:10.3389/fpls.2026.1859834)
Supplement: Supplementary Table 1 — Primer sequences of genes involved in steviol glycoside biosynthesis. [file Table1.docx]

Supplementary Material

# Supplementary Tables

**Supplementary Table S1.** Primer sequences of genes involved in steviol glycoside biosynthesis

| Gene Name | Sequence 5'-3' |
| --- | --- |
| ent-KAH | F-CCATATTCACCATCCGACTTGG |
|  | R-GGGTAGTGAAGATCTCCTTAGC |
| UGT74G1 | F-TGCATGAACTGGTTAGACGATAAG |
|  | R-GCATCCTACTGATTCGTGTGCTA |
| UGT76G1 | F-GCAGCTTACTAGACCACGATC |
|  | R-CTCATCCACTTCACTAGTACTAC |
| 18S rRNA | F-CCGGCGACGCATCATT |
|  | R-AGGCCACTATCCTACCATCGAA |

**Supplementary Table S2.** Physiological parameters at week 4 of *Stevia rebaudiana* plants grown under drought stress conditions.

| WHC (%) | Treatment | Plant Height (cm) | New Shoots (n) | Chlorophyll (SPAD) | LAI (cm) | Biomass (g) | Root (g) |
| --- | --- | --- | --- | --- | --- | --- | --- |
| 100 | C | 17.13±1.30b | 3.55±0.58a | 33.7±1.65a | 5.31±0.67a | 21.01±4.99a | 13.2±2.02a |
|  | W | 10.60±4.72ab | 7.00±3.16a | 29.5±1.55a | 4.50±0.75a | 15.45±8.12a | 10.8±2.76a |
|  | B | 9.63±5.59ab | 16.33±2.52ab | 19.7±3.04a | 5.05±1.77a | 19.59±5.88a | 14.5±0.83a |
|  | WB (1:1) | 13.82±7.32ab | 8.60±7.23a | 30.9±2.63a | 5.50±1.33a | 24.53±5.14a | 18.4±1.34a |
|  | WB (3:1) | 14.95±6.99ab | 10.80±6.98ab | 29.8±1.48a | 5.40±1.57a | 22.10±7.51a | 16.3±2.36a |
|  | WB (1:3) | 7.63±7.50ab | 13.00±3.32ab | 22.4±2.60a | 4.67±1.55a | 23.04±6.05a | 18.6±2.34a |
| 85 | C | 12.18±4.10ab | 4.00±2.94a | 29.0±2.46a | 6.72±0.57a | 26.46±7.54a | 18.6±3.15a |
|  | W | 12.84±2.54ab | 8.80±5.72a | 27.5±3.03a | 7.66±0.96a | 23.58±3.75a | 18.2±2.49a |
|  | B | 10.10±2.34ab | 9.50±3.00a | 22.3±6.07a | 3.37±0.65a | 20.26±8.52a | 16.0±2.18a |
|  | WB (1:1) | 11.32±5.88ab | 7.40±4.98a | 31.8±1.32a | 5.21±0.76a | 18.44±14.48a | 13.6±0.77a |
|  | WB (3:1) | 12.40±3.76ab | 11.40±6.80ab | 29.4±4.62a | 5.76±1.16a | 25.34±13.59a | 18.7±3.74a |
|  | WB (1:3) | 11.20±1.27ab | 4.60±3.44a | 25.5±3.00a | 3.82±0.88a | 14.19±5.71a | 11.8±2.57a |
| 65 | C | 12.28±3.82ab | 6.00±4.74a | 30.4±1.15a | 4.86±0.49a | 20.19±4.92a | 15.3±0.98a |
|  | W | 12.46±6.29ab | 7.80±5.54a | 28.6±4.01a | 5.38±1.94a | 20.51±10.96a | 16.5±4.71a |
|  | B | 7.44±0.38ab | 13.00±6.63ab | 19.4±4.17a | 1.99±0.84a | 18.88±7.49a | 14.6±1.99a |
|  | WB (1:1) | 10.97±3.75ab | 15.60±2.07ab | 25.4±3.28a | 5.67±1.45a | 27.71±9.13a | 20.7±3.25a |
|  | WB (3:1) | 7.60±9.40ab | 10.20±4.66ab | 22.5±5.70a | 3.32±1.52a | 20.46±6.25a | 17.0±2.72a |
|  | WB (1:3) | 13.15±4.71ab | 5.40±2.61a | 31.6±2.21a | 6.04±1.43a | 21.28±8.03a | 17.3±2.65a |
| 45 | C | 10.32±5.65ab | 6.00±3.39a | 28.5±4.03a | 5.32±0.65a | 15.30±5.50a | 11.4±1.78a |
|  | W | 5.14±2.89a | 14.00±11.55ab | 25.5±5.30a | 5.54±1.89a | 16.86±7.88a | 13.5±2.60a |
|  | B | 6.50±2.12ab | 22.20±5.67b | 20.6±3.56a | 3.81±1.27a | 20.39±9.26a | 16.3±3.25a |
|  | WB (1:1) | 7.47±1.29ab | 6.80±4.21a | 23.1±3.87a | 3.40±0.77a | 20.10±4.66a | 17.1±1.66a |
|  | WB (3:1) | 8.98±3.00ab | 10.75±4.43ab | 24.1±2.46a | 5.61±1.76a | 24.00±12.51a | 19.4±4.92a |
|  | WB (1:3) | 10.00±2.00ab | 6.25±5.12a | 24.1±1.73a | 6.17±1.91a | 18.22±4.55a | 13.9±0.91a |

- Data are expressed as Mean ± Standard deviation (SD). Different letters in the same column indicate statistically significant differences among treatments according to Tukey’s multiple comparison test (*p* < 0.05).
- **Supplementary table S3.** Comprehensive Biochemical and Antioxidant Profiles of at week 4 of *Stevia rebaudiana* plants grown under drought stress conditions

| WHC (%) | Treatment | Electrolyte Leakage (%) | TPC (mg/g) | TFC (mg/100 g) | DPPH (%) | ABTS (%) | Gallic Acid (μg/g) | Quercetin (μg/g) |
| --- | --- | --- | --- | --- | --- | --- | --- | --- |
| 100 | C | 11.12±2.71a | 1.26±0.24bcdef | 0.944±0.20ghi | 28.15±9.41cdef | 67.09±5.18ab | 104.30±10.62abc | 101.98±4.22g |
|  | W | 8.32±1.94a | 1.35±0.33cdefg | 0.60±0.10abcdef | 27.93±9.31cdef | 69.79±8.72ab | 108.80±2.64abcd | 6.77±3.23ab |
|  | B | 25.96±3.96a | 1.22±0.22ghi | 0.94±0.16bcdef | 19.53±3.22abc | 65.79±5.03ab | 133.05±4.64bcdefghij | 22.42±0.69cd |
|  | WB (1:1) | 20.51±9.07a | 1.41±0.29efg | 1.03±0.25hi | 28.96±8.62defg | 68.67±5.13ab | 102.55±4.91abc | 22.24±1.46cd |
|  | WB (3:1) | 14.27±4.14a | 1.25±0.34fghi | 0.82±0.16bcdef | 28.03±4.95bcdef | 72.66±3.90ab | 114.49±5.59abcde | 25.73±2.65cd |
|  | WB (1:3) | 31.44±9.81a | 1.13±0.09abcde | 0.53±0.13abcde | 15.42±2.46a | 65.22±6.62ab | 157.23±20.84defghij | 21.97±2.80cd |
| 85 | C | 16.34±5.63a | 1.01±0.21 ab | 0.61±0.31bcdef | 21.42±7.14abcde | 69.08±9.05ab | 171.48±7.62hijk | 54.86±5.06e |
|  | W | 13.36±3.61a | 1.01±0.15ab | 0.47±0.16ab | 20.02±5.49abcd | 70.50±3.14ab | 162.60±2.44efghijk | 19.33±1.97bc |
|  | B | 16.54±4.83a | 1.06±0.18abcd | 0.98±0.30hi | 32.46±9.21fgh | 65.17±1.21ab | 82.80±1.55ab | 67.20±2.21f |
|  | WB (1:1) | 10.21±2.89a | 1.26±0.19hi | 1.00±0.22bcdef | 30.38±8.62efgh | 70.84±2.80ab | 178.23±7.61ijk | 23.13±1.62cd |
|  | WB (3:1) | 17.54±6.15a | 1.09±0.20abcde | 0.52±0.10 abcde | 26.17±4.60ab | 68.80±1.56ab | 119.84±5.98abcdef | 26.71±2.19cd |
|  | WB (1:3) | 16.78±6.38a | 1.66±0.43g | 0.77±0.20cdefgh | 37.81±1.26gh | 69.32±8.95ab | 187.11±36.50ijk | 21.86±0.62cd |
| 65 | C | 10.52±3.57a | 1.20±0.34bcdef | 0.56±0.19abcdef | 19.84±8.27abc | 66.66±11.44ab | 137.53±7.71cdefghi | 137.11±6.24h |
|  | W | 8.96±1.25a | 0.86±0.12a | 0.31±0.23a | 15.65±5.64a | 62.79±5.02a | 168.43±8.26fghijk | 5.78±0.92a |
|  | B | 17.37±4.79a | 0.99±0.03ab | 0.81±0.20efghi | 37.20±6.84gh | 74.06±1.74bc | 68.53±4.21a | 24.91±1.04cd |
|  | WB (1:1) | 11.97±5.03a | 1.10±0.35ghi | 0.91±0.15abcde | 30.38±9.01ab | 71.05±6.22ab | 184.56±10.88jk | 23.82±2.48cd |
|  | WB (3:1) | 23.64±7.27a | 1.08±0.08abcde | 0.74±0.15abcde | 17.30±5.33ab | 65.34±3.30ab | 182.91±1.93hijk | 31.86±3.51cd |
|  | WB (1:3) | 23.51±9.32a | 1.50±0.02fg | 1.10±0.19i | 31.57±4.57fgh | 68.40±6.06ab | 171.50±10.39ghijk | 26.08±2.25cd |
| 45 | C | 10.78±1.42a | 1.39±0.06defg | 0.66±0.17bcdefg | 22.41±6.58abcde | 73.36±5.17bc | 97.36±4.83abc | 63.48±7.03ef |
|  | W | 17.30±5.49a | 1.07±0.21abcd | 0.67±0.18bcdefg | 18.37±2.41ab | 64.31±4.07ab | 121.76±5.22abcdef | 20.59±0.67cd |
|  | B | 19.37±5.09a | 1.03±0.09abc | 0.78±0.04defgh | 38.20±2.60h | 73.44±0.71bc | 122.90±2.70abcdefgh | 19.03±0.58abcd |
|  | WB (1:1) | 33.15±10.5a | 1.02±0.16ab | 0.48±0.36abc | 18.28±4.64cdef | 83.37±10.76c | 206.42±96.65k | 26.29±19.53cd |
|  | WB (3:1) | 15.01±4.22a | 1.25±0.10bcdef | 0.56±0.10abcdef | 17.53±0.52abc | 70.69±1.98ab | 134.70±6.11 bcdefghij | 18.99±0.53abcd |
|  | WB (1:3) | 15.47±5.19a | 1.00±0.13ab | 0.50±0.13abcd | 18.41±5.19ab | 74.32±17.48bc | 124.26±15.82abcdefg | 33.24±6.79d |

- Data are expressed as Mean ± Standard deviation (SD). Different letters in the same column indicate statistically significant differences among treatments according to Tukey’s multiple comparison test (*p* < 0.05).
